# Effects of Dielectrophoresis on Growth, Viability and Immuno-reactivity of *Listeria monocytogenes*

**DOI:** 10.1186/1754-1611-2-6

**Published:** 2008-04-16

**Authors:** Liju Yang, Padmapriya P Banada, Arun K Bhunia, Rashid Bashir

**Affiliations:** 1 Biomanufacturing Research Institute and Technology Enterprise (BRITE), and Department of Pharmaceutical Sciences, North Carolina Central University, Durham, NC 27707, USA; 2 Molecular Food Microbiology Laboratory, Department of Food Science, Purdue University, West Lafayette, IN 47907, USA; 3 Birck Nanotechnology Center, School of Electrical and Computer Engineering, Weldon School of Biomedical Engineering, Purdue University, West Lafayette, IN 47907, USA; 4 Now at Center for infectious diseases, Department of Medicine, New Jersey Medical school-UMDNJ, 185 S. Orange Ave, Newark, NJ 07130, USA; 5 Now at Department of Electrical and Computer Engineering and Department of Bioengineering, Micro & Nanotechnology Laboratories, University of Illinois, Urbana-Champaign, Urbana, IL 61801, USA

## Abstract

Dielectrophoresis (DEP) has been regarded as a useful tool for manipulating biological cells prior to the detection of cells. Since DEP uses high AC electrical fields, it is important to examine whether these electrical fields in any way damage cells or affect their characteristics in subsequent analytical procedures. In this study, we investigated the effects of DEP manipulation on the characteristics of *Listeria monocytogenes *cells, including the immuno-reactivity to several *Listeria-*specific antibodies, the cell growth profile in liquid medium, and the cell viability on selective agar plates. It was found that a 1-h DEP treatment increased the cell immuno-reactivity to the commercial *Listeria *species-specific polyclonal antibodies (from KPL) by ~31.8% and to the C11E9 monoclonal antibodies by ~82.9%, whereas no significant changes were observed with either anti-InlB or anti-ActA antibodies. A 1-h DEP treatment did not cause any change in the growth profile of *Listeria *in the low conductive growth medium (LCGM); however, prolonged treatments (4 h or greater) caused significant delays in cell growth. The results of plating methods showed that a 4-h DEP treatment (5 MHz, 20 Vpp) reduced the viable cell numbers by 56.8–89.7 %. These results indicated that DEP manipulation may or may not affect the final detection signal in immuno-based detection depending on the type of antigen-antibody reaction involved. However, prolonged DEP treatment for manipulating bacterial cells could produce negative effects on the cell detection by growth-based methods. Careful selection of DEP operation conditions could avoid or minimize negative effects on subsequent cell detection performance.

## Background

*Listeria monocytogenes *is considered as one of the most hazardous, potentially life-threatening, human foodborne pathogens. It can contaminate many food products, such as milk, cheese, ice cream, raw vegetables, poultry products, and meats. The Centers for Disease Control and Prevention (CDC) estimates that there are 2,500 illnesses with 500 deaths associated with listeriosis in the United States each year [1]. The development of rapid, sensitive, simple and cost effective methods to detect this pathogen is extremely important in implementing an effective response to the prevention of foodborne diseases. Conventional microbiological methods are time-consuming, largely because they require several enrichment and separation steps (e.g. pre-enrichment, selective enrichment) to grow cells to detectable concentrations. Many recently developed rapid methods have sought to accelerate or eliminate traditional growth-based enrichment steps by using newly discovered concentration or separation methods that are not limited by bacterial growth. These methods include membrane filtration, magnetic separation, dielectrophoresis, and electrophoresis to concentrate bacteria cells [2]. Among these, dielectrophoresis (DEP) has been proven especially suitable for manipulation, concentration, and separation of biological cells in micro-scaled devices, and has great potential to be integrated with various detection methods [3,4].

DEP is the electrokinetic motion of dielectrically polarized particles in a non-uniform AC electrical field due to the unbalanced force of the electrical field on the particle's induced dipole moment [5]. The dielectrophoretic force, *F*_DEP_, acting on a dielectric spherical particle suspended in a medium in an electrical field can be approximated as Eq. 1 [6-9],

FDEP=2πr3ε0εmRe⁡[fCM(ω)]∇Erms2

where, r is the particle radius, *ε*_0 _is the vacuum dielectric constant, *ε*_*m *_is the permittivity of the suspending medium, *E*_*rms *_is the root mean square value of the electrical field, and Re[*f*_*CM*_(*ω*)]the real part of the Clausius-Mossotti factor, given by

$$f_{\text{CM}}(\omega )=\frac{\varepsilon_{\text{p}}^{*}-\varepsilon_{\text{m}}^{*}}{\varepsilon_{\text{p}}^{*}+2\varepsilon_{\text{m}}^{*}}$$

where, $\varepsilon_{p}^{*}=\varepsilon_{p}-j\sigma_{p}/\omega$ and $\varepsilon_{m}^{*}=\varepsilon_{m}-j\sigma_{m}/\omega$ are complex permittivities of the particle and the medium respectively, with *σ *the conductivity, *ε *the permittivity, *ω *the angular frequency of the applied electrical field, and $j=\sqrt{-1}$. The frequency dependence of Re[*f*_*CM*_(*ω*)] indicates that the force acting on the particle varies with the frequency. Depending on the relative polarizability of the particle with respect to the surrounding medium, the particle will be induced to move either towards a region where the electrical field gradients are the strongest (Re[*f*_*CM*_] > 0) (positive DEP), or towards a region where the electrical field gradients are the weakest (Re[*f*_*CM*_] < 0) DEP (negative DEP).

As most biological cells behave as dielectrically polarized particles in a non-uniform electrical field, they can be manipulated by DEP for various applications. Well demonstrated applications of DEP for manipulation of cells are the separations of different types of cells based on the differences in the dielectrical polarizabilities among these cell types [3]. Examples of these applications include the separation of viable and nonviable yeast cells [10,11], cancer cells and normal cells [12-15], CD34+ cells and blood stem cells [16], individual neurons [17], the trapping of viruses from fluid [18], and the separation and detection of bacterial cells [3,19-23].

DEP has been employed to manipulate *Listeria *cells for separation, concentration, and/or detection purposes. Li and Bashir [9] reported a DEP-based separation method to separate live and heat-killed *Listeria monocytogenes *cells in a static solution on microfabricated interdigitated electrodes. The separation was based on the large difference in dielectrical properties between live and dead cells. DEP has afforded the development of advanced lab-on-a-chip devices by integrating its multi-functions (concentration and separation) with different analytical detection technologies [7]. Gomez et al. [24] developed the on-chip impedance microbiology to detect *Listeria *cells. Live *Listeria *cells in the fluid were successfully concentrated into an ultra-small volume (400 pl) in a micro-device by DEP, and were followed by impedance detection of bacterial growth. The concentration factor of the chip was between 10^4 ^to 10^5 ^when the cells in an original sample volume of 40 μl were concentrated into the 400 pl chamber. Such a DEP concentration step eliminated the need for lengthy bacterial population enrichment steps using conventional cell culture methods, and drastically reduced the total assay time. Yang et al. [25] employed DEP to collect and concentrate *Listeria monocytogenes *cells in a microfluidic channel and combined it with antibody-based capture of cells in the microfluidic device. The device utilized an interdigitated microelectrode embedded in the microfluidic channel for DEP collection of cells. Monoclonal anti-*Listeria monocytogenes *antibodies were immobilized on the microelectrode surface which provided selective capture of *Listeria monocytogenes *cells. DEP served to concentrate *Listeria *cells at the locality of the electrodes, and to make cells in close contact with antibodies immobilized on the channel and electrode surfaces, which in combination dramatically improved the capture efficiency of antibodies to cells in the microfluidic device. Such a DEP microfluidic device was particularly useful for trapping and detecting low concentrations of cells.

DEP has also been widely used to characterize and/or detect other microorganisms. Lapizco-Encinas et al. [26] reported a DEP method to concentrate and remove microbes (*Bacillus subtilis *spores, Tobacco Mosaic Virus, *Escherichia coli *cells) from water. Suehiro et al. [23] combined DEP with the impedance method to selectively detect *E. coli*. After dielectrophoretic trapping of bacteria, antibodies were added to agglutinate target bacteria. Agglutinated bacteria whose apparent size increased experienced greater DEP forces and were thus trapped in the gap of the electrodes, while other non-agglutinated non-target bacterial cells were washed out in the wash steps. They also immobilized anti-*E. coli *antibodies onto the electrode surfaces so that only antibody-specific bacteria would be bound to the electrode. Cells were collected by DEP in the gaps between the electrodes, and then impedance changes due to the captured cells were monitored [27]. The same group reported an improved DEP impedance method to detect *E. coli *by combining DEP with electropermeabilization (EP) [28]*. E. coli *cells in suspension were captured onto an interdigitated microelectrode array by positive DEP. EP was then performed by applying a high AC electrical field to the trapped bacteria which led to intracellular ion release through damaged cell membranes, and caused an increase in conductance. Using this method, 10^2 ^cfu/ml of *E. coli *was detected in 3 h.

These studies have demonstrated that DEP is a useful technique to develop advanced multifunctional detection methods for rapid detection of microorganisms. In these detection methods, cells are manipulated by DEP prior to various detection steps, which may involve antibody-based immunoreaction, bacterial growth/metabolism, or DNA analysis. Since cells are exposed to AC electrical fields during DEP manipulation, it is imperative to examine whether such electrical field exposure induces undesirable effects on the cells which may affect the analytical performance in these subsequent detection procedures. A number of studies have shown that pulse and DC electrical fields applicable to electroporation and cell fusion can seriously alter the characteristics of mammalian cells. These effects include alteration in cell membrane potentials and cell membrane structures [29-31], cell deformation [29,31-33], and increases in cell membrane permeability [32-34]. Wang et al. [35] studied the effects of AC field exposure on the viability and proliferation of mammalian cells in DEP manipulation, and found that extended lag phases in cell growth following electrical field exposure were due to toxic reactions of cells with electrochemical species produced at the electrodes. However, other studies have reported that DEP treatment has no serious effect on cells. For instance, Huang et al. [4] found that DEP forces had little effect on cell survival or stress by analyzing the expression of the stress-related gene c-fos. A number of studies showed that DEP did not cause major damages to various types of cells, including erythrocytes [36,37], yeast cells [11], and CD34+ cells [16]. Some other studies have reported that DEP treatment's effect largely depends on the experimental conditions. Wang et al. [35] and Altomare et al. [38] examined the effects of experimental DEP on tumor cell growth kinetics and their ability to undergo differentiation. They concluded that DEP induced effects on tested tumor cells depended on the buffer used in the experiments. However, these studies have been mostly focused on mammalian cells; little has been done to study the DEP effects on bacterial cells. In our previous study, we found that the expression of *L. monocytogenes *antigens that are specific for C11E9 monoclonal antibody increased ~2–3 folds after *Listeria *cells were manipulated by DEP [25].

In this study, we investigated the effects of DEP on the immuno-reactivity of *Listeria monocytogenes *cells to several anti-*Listeria *antibodies using enzyme-linked immunosorbant assays (ELISA), on the cell growth profile in liquid medium, and on the cell viability on selective agar plates. These cell characteristics are commonly used in various detection techniques, such as antibody-based tests and growth-based tests, to detect bacterial cells. The results from this study are useful for the selection of experimental DEP conditions for concentration and manipulation of *Listeria *cells to avoid or minimize possible negative effects in integrated detection methods.

## Methods

### Bacteria cultures and media

*Listeria monocytogenes *V7 culture, a milk isolate of serovar 1/2a was grown in brain heart infusion (BHI) broth at 37°C for 16–18 h in a shaker incubator with a constant agitation at 140 rpm. The cells were pelleted by centrifugation (Eppendorf, Westbury, NY)) at 6,000 × g for 5 min and resuspended in sterilized deionized (DI) water. The cell numbers were determined by surface plating 0.1 ml of appropriate dilutions onto modified oxford agar (MOX) (Difco, Sparks, MD). Colonies were counted after incubation of the plates at 37°C for 24 h. The concentration of cells in the culture averaged about 10^9 ^colony forming units per milliliter (cfu/ml).

*L. monocytogenes *cells were stained with 3,3'-dihexyloxacarbocyanine iodide (DiOC_6_(3)) dye (green) (Molecular Probes, Eugene, OR) for visualization purposes under a fluorescence microscope. All stained bacteria suspensions were washed and centrifuged with DI water for 4 to 5 times to remove excess dye molecules. Serial dilutions were prepared in DI water for further applications when needed.

### Dielectrophoresis (DEP) device and treatment

**Figure 1:**
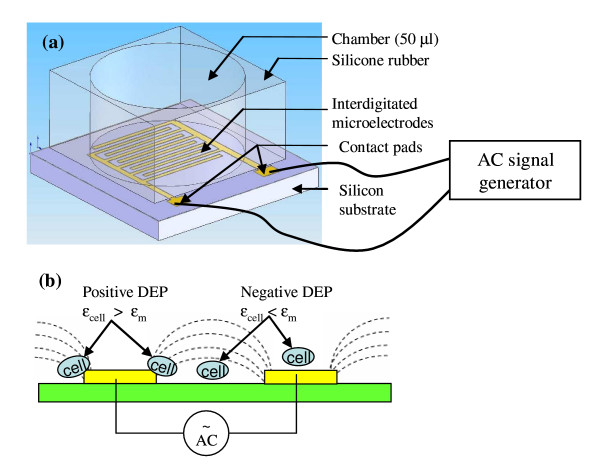
**(a) The device used in this study for DEP treatments of *Listeria *cells. It consists of a chamber formed by silicone rubber and a set of interdigitated microelectrodes at the floor of the chamber.** The interdigitated electrode has a total of 40 pairs of finger electrodes, each measuring 25 μm of electrode width and 25 μm of space. (b) Schematic of the non-uniform electrical field generated by the interdigitated microelectrodes, and the positive and negative DEP for bacterial cells in this electrical field. The electrical field has its maximum in gradients and strength at the edges of finger electrodes and its minimum at the centers of the finger electrodes. Positive DEP moves cells to the edges of these finger electrodes where the electrical field is stronger, while negative DEP moves cells towards the centers of the finger electrodes between the electrodes where the electrical field is weaker.

Fig. 1a shows the device for DEP treatment. It consists of an array of interdigitated microelectrodes on a flat silicon substrate and a chamber (50 μl capacity) right above the electrode formed by silicone rubber. The interdigitated array (IDA) microelectrodes were fabricated on 4" silicon wafers with a (100) surface and a thickness of 500 μm using a similar photolithographic procedure in our previous report [24]. The wafers were thermally oxidized to create a 2000 Å layer of silicon dioxide. On top of the oxide, the IDA electrodes were patterned and deposited by sputtering of platinum to a thickness of 1000 Å. The IDA has a total of 40 pairs of platinum finger electrodes each measuring 25 μm of electrode width and 25 μm of space.

For DEP treatment, 50 μl of *L. monocytogenes *cells (10^8^–10^9 ^cfu/ml) was introduced into the chamber, and a glass cover was used to cover the chamber to prevent evaporation. The chamber was allowed to sit for at least 2 h at room temperature to let the bacterial cells settle on the surfaces of the electrodes. An Agilent 33120A arbitrary waveform generator (Agilent Technologies, Inc., Palo Alto, CA) was used to apply a sinusoidal voltage to the DEP electrodes at 20 Vpp with different frequencies for desired test periods. For control experiments, the same number of cells were processed in the same way but no DEP voltage was applied. Then the DEP-treated and -untreated cells were examined in parallel for their reactivity to different antibodies using ELISA methods, their growth profile using real time pH measurements, and their viability using conventional plating methods.

### ELISA tests for the immuno-reactivity of *Listeria *cells to different antibodies

Aliquots of 50 μl (10^9 ^cfu/ml) of DEP-treated and -untreated *Listeria *cells were dispensed into the wells of a flat-bottomed 96-well microtiter plates (1B Immulon, ThermoLabsystems, Milford, MA). The plate was incubated overnight at 4°C and the wells were washed with phosphate buffered saline (PBS) containing 0.5% Tween 20 (PBST, pH 7.4) to remove unbound cells. Mouse anti-*Listeria *monoclonal antibody C11E9 (MAb-C11E9) (0.02 mg/ml) [39], rabbit polyclonal antibody Lm404 (PAb Lm404), rabbit polyclonal antibody C639 (PAb C639) [40]; and horseradish peroxidase (HRP) conjugated anti-*Listeria *polyclonal antibody from KPL (cat# 04-90-90, KPL Inc., Gaithersburg, MD) were used to study the reactivity of the cells to these antibodies. C11E9 belongs to the IgG2b subclass and reacts with 5 different surface antigens with a major reactive antigen being the 66-kDa N-acetylmuramidase [41]. PAb Lm404 and PAb C639 react with Internalin B (inlB) and Actin polymerization protein A (ActA) on *Listeria *cell surfaces [40]. These antibodies were added to the bacteria-coated wells, and the plate was incubated for 1 h at 37°C, with constant shaking. After washing three times with PBST to remove unbound antibodies, 100 μl (1:5000) of HRP-conjugated anti-mouse (for C11E9) or anti-rabbit (for PAb Lm404 and PAb C639) secondary antibody (Jackson Immuno Research Laboratories, Westgrove, PA) was added to each well. After washing three times with PBST, the substrate O-phenylene diamine (OPD) (Sigma, St. Louis, MO) was added to those wells to develop color products for absorbance measurements. The wells containing KPL anti-*Listeria *antibody were developed by directly adding the OPD substrate solution, since the KPL antibody was HRP conjugated. The reactions were stopped after 15 min by adding 100 μl 0.1 M HCl into each well. The absorbance of each well was read at 490 nm using an ELISA reader (Bio-Rad, Hercules, CA).

### Bacterial growth detection using real time pH measurements

The pH measurement procedure was similar to that of our previous study [42]. Aliquots of 50 μl of DEP-treated or -untreated sample were introduced into 15 ml of the BioV LCGM™ growth medium (BioVitesse, Inc., San Jose, CA) [43] in a 50 ml centrifuge tube (Becton Dickinson Labware, Franklin Lakes, NJ). The tube was then placed in an incubator (Lab-Line Instruments, Inc. Melrose Park, IL) and kept at 37 ± 0.5°C. The pH of the sample was measured using a pH probe (Serial No. JC05708, Jenco, San Diego, CA) immersed in the medium. pH data was collected every 5 min during the growth of *L. monocytogenes *within a total testing period of 18 h. pH growth curves were obtained by plotting the pH value as a function of growth time.

### Cell viability tests using plating methods

The DEP-treated or -untreated cells were serially diluted with DI water. The viable cell numbers were determined by surface plating appropriate dilutions onto MOX agar. MOX agar is a selective growth medium for *Listeria *cells, to which antibiotic agents are added to suppress the growth of other competing microflora. Thus, in the presence of these selective agents, the injured or stressed cells are unable to grow and form characteristic colonies on this agar. The difference in the number of colony-forming units of DEP-treated and -untreated samples indicates the number/percentage of the injured or stressed cells due to cell exposure to the DEP electrical field.

### Imaging

The bright field and fluorescence images were taken on a Nikon ECLIPSE E600FN fluorescence microscope (Japan) attached with a CCD camera (Pixera, Los Gatos, CA). Fluorescence images were taken using the FITC (Fluorescin isothiocyanate) – specific filter.

### Field emission scanning electron microscopy (FESEM)

*Listeria *cells collected on the chip by DEP were air-dried overnight at room temperature. The chip was directly imaged using a Hitachi S 4800 FESEM microscope (Tokyo, Japan) without coating. Acceleration voltage was kept constant at 2.0 kV. Images were acquired digitally using Quartz PCI v.7 software (Hitachi High-Technologies Canada, Inc. Resdale, Ontario, Canada).

### Statistical analysis

Significant differences were determined by the standard ANOVA and Tukey's-test, using SAS 9.1 software (SAS Institute Inc., Cary, NC).

## Results and Discussion

### DEP manipulation of *L. monocytogenes *on interdigitated microelectrodes

The main component of any dielectrophoresis system is formed by the electrodes on which the AC electrical field is applied. The electrode configuration which determines the generated non-uniform electrical field is one of the important factors for efficient dielectrophoretic collection of biological cells. Many different electrode configurations have been reported to realize desirable cell trapping in micro-scale dielectrophoresis systems. Electrode structures made from thin wires, such as cone-plate electrodes [44,45], simple pin-plate structures [46], and four-pole electrodes [47], have been used for dielectrophoretic characterization of cells. More recently, different microfabricated electrodes have played a major role in dielectrophoresis devices. Examples include planar polynomial microelectrodes arrays for trapping different types of cells at different locations by using positive and negative DEP [19], various three-dimensional microelectrode arrays for cell position, more complex 3-D extruded quadrupole structures for trapping single cells and particles [48-50], and a novel "points-and-lid" microelectrode system for DEP registration of single mammalian cells to a microelectrode [51].

The planar interdigitated array (IDA) microelectrode (shown in Fig. 1a) is perhaps the simplest electrode structure that has been used successfully in DEP manipulation of bacteria cells. Such a microelectrode can be readily integrated with micro-fluidic channels. Fig. 1b shows the schematic of the electrical field generated by the IDA electrodes. The electrical field has its maximum gradient and strength at the edges of the digit electrodes and the minimum at the centers of the digit electrodes and the gaps between the digit electrodes. When cells are placed in this non-uniform electrical field, they will experience positive DEP or negative DEP depending on the DEP operation frequency and the relative polarizability of the cells with respect to the medium (Fig. 1b). Positive DEP forces will direct cells to the edges of these digit electrodes where the electrical field is stronger, while negative DEP will move cells towards the centers of the digit electrodes or the gaps between the electrodes where the electrical field is weaker.

**Figure 2:**
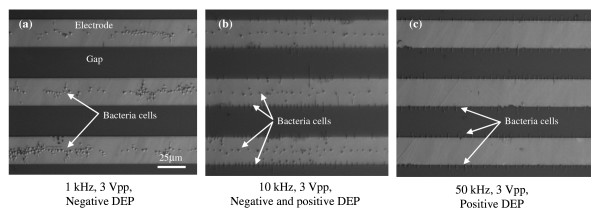
**Microscopic images of *Listeria monocytogenes *exhibiting negative and positive DEP at the interdigitated microelectrode.** (a)*Listeria monocytogenes *cells exhibit negative DEP at 1 kHz and 3 Vpp; they are collected at the centers of the finger electrodes. (b) Some of the cells exhibit negative DEP, while others exhibit positive DEP, at 10 kHz and 3 Vpp; Cells are collected at both the centers of the finger electrodes and the edges of the finger electrodes. (c) As frequency increases to 50 kHz (at 3 Vpp), all the cells exhibit positive DEP and are collected at the edges of the finger electrodes.

Fig. 2 shows the microscopic images of *Listeria *cells experiencing (a) negative DEP at 1 kHz and 3 Vpp, (b) both negative and positive DEP at 10 kHz and 3 Vpp, (c) positive DEP at 50 kHz and 3 Vpp. *Listeria *cells in DI water suspensions exhibit negative DEP at low frequencies (~1 kHz or lower); as the frequency increases, the cells begin to exhibit positive DEP. At 10 kHz, some of the cells exhibit negative DEP, while others exhibit positive DEP, due to the slight variations among these individual cells. When the frequency increases to 50 kHz and higher, all the cells exhibit positive DEP. This frequency-dependent positive and negative DEP of *Listeria *cells implies that the frequency of the DEP electrical field affects the dielectrical properties of cells, thus determining whether they exhibit positive or negative DEP. According to Eq.1 and Fig. 1b, cells exhibiting positive DEP have their permittivity higher than that of medium, while cells exhibiting negative DEP have their permittivity lower than medium permittivity. Like all biological cells, bacterial cells consist of adjacent structures of materials that have very different electrical properties. The cell membrane consists of a lipid bilayer containing many proteins where the lipid molecules are oriented with their polar groups facing outwards into the aqueous environment and their hydrophobic hydrocarbon chains pointing inwards to form the membrane interior. The inside of a cell, the cytoplasm, is complex and contains many dissolved charged molecules. While the cell membrane is highly insulating, the interior of the cell is highly conductive. The conductivity of the cell membrane is around 10^-7 ^S/m, whereas the conductivity of the interior of a cell can be as high as 1 S/m [3].

For DEP manipulation of biological cells, cells are usually suspended in low conductivity buffers. Buffers for suspending mammalian cells usually contain different sugars, such as a Tris-Boric acid-EDTA buffer supplemented with 250 mM sucrose having a conductivity of ~10 μS/cm [14], and another buffer consisting of 8.5% (w/v) sucrose plus 0.3% (w/v) dextrose having a conductivity of 50 μS/cm [52]. Deionized (DI) water is often used for suspending bacterial cells for DEP manipulation [9,24-26]. In this study, we used DI water to suspend *Listeria *cells for all the experiments. DI water has its conductivity in a range from 1–2 μS/cm to about 10–15 μS/cm. In DEP manipulation, most likely, at low frequencies, the electrical field is mainly dropped across the outermost membranes of the cells. The cells behave as poorly conductive spheres [6,9]. As the frequency increases, the applied field gradually penetrates into the cells. The cells then behave as more conductive spheres with high permittivity of the cell interior [9]. At low frequency (*ω *<<*σ/ε*), Eq. 2 can be approximated by [53]:

*f*_*CM *_= (*σ*_*p *_- *σ*_*m*_)/(*σ*_*p *_+ 2*σ*_*m*_)

While at high frequency (*ω *>> *σ/ε*):

*f*_*CM *_= (*ε*_*p *_- *ε*_*m*_)/(*ε*_*p *_+ 2*ε*_*m*_)

Thus it is possible that cells exhibit negative DEP at low frequency if *σ*_p _<*σ*_m_, (Re[*f*_CM_] < 0 in Eq. 1) and positive DEP at high frequency if *ε*_p _> *ε*_m _(Re[*f*_CM_] < 0 in Eq. 1). It is also known that in positive DEP, cells are collected at the electrode edges where electrical field is the strongest. One can imagine that cells experiencing positive DEP at higher frequencies would more likely be affected by the electrical field compared with cells experiencing negative DEP at lower frequencies.

In practice, when DEP is used to concentrate or capture cells in micro-fluidic devices for subsequent detection, higher frequencies and voltages are usually required to achieve efficient capture of bacterial cells from the flow. Gomez et al. [24] reported that an AC signal at 3 MHz and 20 Vpp were needed to maximize the DEP forces acting on *L. innocua *cells to capture them from the Luria-Bertani broth. Yang et al. [25] reported that *L. monocytogenes *cells in the flow of DI water were captured by DEP at a frequency of 1 MHz and 20 Vpp. Verduzco-Luque et al. [54] used DEP to manipulate cells to make biofilms. They showed that *Saccharomyces pombe *cells suspended in DI water were oriented at right angles to the electrical field at 80 MHz and 3.5 Vpp. Patterning of 3T3 mouse fibroblast cells suspended in a 480 mM mannitol solution into the gaps between microelectrodes was achieved by positive DEP at 1 MHz and 20 Vpp [55]. Suehiro et al. [23] used IDA microelectrodes to trap *E. coli *cells using DEP at 100 kHz and 5 Vpp onto the electrode surfaces for impedance measurements. Considering the DEP parameters used in these studies and the effectiveness of DEP manipulation of *Listeria *cells in microfluidic and/or non-fluidic devices, we selected DEP at 5 MHz and 20 Vpp for manipulation of *Listeria *cells in microdevices, and examined the effects of DEP on the immuno-reactivity of *Listeria *cells to several *Listeria-*specific antibodies, on cell growth profile in LCGM medium, and on cell viability on MOX agar plates.

**Figure 3:**
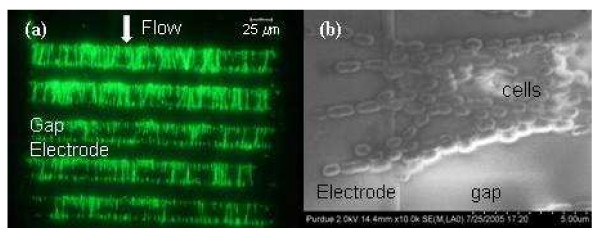
**(a) The fluorescence image of *Listeria *cells collected by positive DEP at 5 MHz and 20 Vpp in a microfluidic channel with the interdigitated microelectrode at the channel floor(40 pairs of finger electrodes, 25 μm of electrode width and 25 μm of space, the channel was ~250 μm wide and 20 μm deep).** Green dots are *Listeria *cells stained with DiOC_6_(3). (b) The FESEM image of *Listeria *cells lined up between the edges of the finger electrodes in the microfluidic channel by positive DEP at 5 MHz and 20 Vpp.

Fig. 3a shows a fluorescence image of *Listeria *cells that were collected in a microfluidic channel by DEP at 5 MHz and 20 Vpp. The cells were trapped at the edges and the gaps of the electrodes and tended to align as a "pearl chain" parallel to the electrical field (green dots are *Listeria *cell stained with DiOC_6_(3)). Fig. 3b presents the FESEM picture of *Listeria *cells in the DEP chamber, clearly showing the "pearl chain" of *Listeria *cells that link the edges of the finger electrodes. It can also be seen that the maximum value of the polarization occurs at the two ends of the rod shaped cells at the experimental DEP condition (5 MHz and 20 Vpp). It is reported the dipole moment of a cell induced by an electrical field can be represented by the generation of equal and opposite charges (+q and -q) at the cell boundary. The magnitude of the induced charge is small, equivalent to around 0.1% of the net surface charge normally carried by cells [56]. Similar to other electrical field treatments such as electroporation and electrofusion, the DEP electrical field acts directly on charges, including free ions, dipoles, ionic and dipolar and polarizable groups. When the electrical field is strong enough, it is possible that molecules and molecular organization such as membranes undergo structural rearrangement. It is known that, in electroporation and electrofusion, direct field effects on the membrane structure are of minor extent. Instead, the interfacial polarization leads to the amplified transmembrane electrical field which actually induces the major structure rearrangement [57]. Regardless of the direct electrical field effects or the induced transmembrane electrical field, it is clear that the charge distribution of the cell under DEP treatment is different from its natural state. This unnatural state condition may induce undesirable effects on the cells which could affect the analytical performance in subsequent detection procedures.

### DEP effects on *Listeria*'s immuno-reactivity to antibodies

Many rapid methods for bacteria detection, such as traditional ELISA or derived ELISA methods, and more recently developed biosensor methods, use an antibody-cell bioaffinity reaction in a sandwich immunoassay format, which involves the formation of immuno-complexes consisting of immobilized antibodies, captured target bacteria and enzyme-labeled antibodies. Most microchip-based methods that use DEP as a concentration step use antibodies to selectively capture target bacterial cells. Therefore, it is logical to examine whether DEP treatment affects the immuno-reactivity of the cells to those antibodies. In our previous study, we found that the number of C11E9 monoclonal antibody binding sites on *Listeria *cells increased from ~5 binding sites per cell to ~10 binding sites per cell upon the DEP treatment when analyzed by SEM [25].

In this study, using ELISA methods, we examined the reactivity of DEP-treated and -untreated *Listeria *cells to monoclonal C11E9 antibody, polyclonal anti-Internalin B (InlB) antibody, polyclonal anti-Actin polymerization protein A (ActA) antibody, and a commercial polyclonal anti-*Listeria *antibody (KPL). InlB and ActA are two major virulence proteins in *L. monocytogenes *that are expressed on the cell surface. These proteins are required for *L. monocytogenes*' entry and intracellular movement inside eukaryotic cells, respectively, and are credible targets for detection of pathogenic *L. monocytogenes*. Previous studies have shown that the reactivity of *Listeria *cells to these antibodies was affected by changes in environmental conditions [40,41,58,59], thus influencing the performance of detection of *Listeria *cells using these antibodies. For example, Geng et al. [41] reported that the reactivity of *L. monocytogenes *cells to a polyclonal antibody and the monoclonal C11E9 antibody was related to the types of growth medium in which the cells were grown. *L. monocytogenes *subjected to stress (acid, cold, heat, and salt) and then grown in a buffered *Listeria *enrichment broth (BLEB) had the greater immuno-reactivity to anti-*Listeria *polyclonal antibody, while those grown in *Listeria *repair broth (LRB) had the greater immuno-reactivity to MAb C11E9. They also found that heat or osmotically stress environments reduced the reactivity of *L. monocytogenes *cells to MAb C11E9 and EM-7G1 antibodies [58], thus affected the detection of *L. monocytogenes *using these antibodies. Therefore, understanding the influence of DEP on the immuno-reactivity of *Listeria *cells is essential to the selection and integration of the best detection antibodies for further applications.

**Figure 4:**
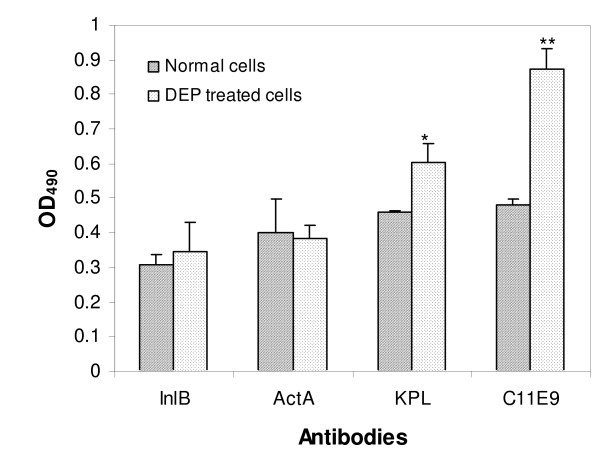
The ELISA results for the immuno-reactivity of DEP treated and untreated *Listeria *cells to PAb Lm404 (anti-InlB), PAb C639 (anti-ActA), anti-*Listeria *species antibody from KPL (Gathersburg, MD) and C11E9 monoclonal antibody. Cell concentrations were about 10^9 ^cfu/ml. Statistically significant difference was observed at P > 0.0002 (*) and P > 0.009 (**).

Fig. 4 shows the results of ELISA tests for DEP-treated and -untreated *Listeria *cells reacted with PAb Lm404 (for InlB), PAb C639 (for ActA), PAb from KPL, and MAb C11E9. The cells were treated with DEP at 5 MHz and 20 Vpp for 1 h. DEP-treated cells did not show significant difference in the absorbance from the immuno-reaction with PAb Lm404 (P > 0.64) and PAb C639 (P > 0.4), indicating that DEP treatment did not affect the reactivity of *Listeria *cells to PAb Lm404 and PAb C639. This implied that DEP treatment did not cause changes in the immuno-reactivity of InlB and ActA on *Listeria *cell surfaces. However, as shown in Fig. 4, the immuno-reactions of DEP-treated *Listeria *cells toward KPL commercial anti-*Listeria *antibody (P > 0.0002) and C11E9 monoclonal antibody (P > 0.009), show significant increases in their absorbance (31.8% and 82.9%, respectively), compared to DEP-untreated cells. These results indicated that DEP treatment did increase the reactivity of *Listeria *cells to the combination of a variety of antigens that bind to KPL polyclonal antibody, and to antigens that are specific to C11E9 monoclonal antibody. It is known that the increased immuno-reactivity is resulted from the increased expression levels of those antigens that are specific to the antibodies [41,58]. A possible explanation for the increased immuno-reactivity of *Listeria *cells to these antibodies due to DEP is that the process of polarization of the cells due to the applied DEP electrical field might cause micro-changes in the membrane structure, causing the epitopes of the antigens on the cell membrane to be more exposed at the cell surface and to become more accessible to the antibodies. The transient membrane permeability increase in electroporation and electrofusion indicates that the external electrical field causes the transient membrane reversible "openings" – pores or cracks which reseal after pulsing [57]. When *Listeria *cells are treated in the high frequency DEP field, they may experience a process similar to the transition to electropores, during which lipid molecules move rapidly away from the pore center, and the remaining bilayer lipids have to change position relative to the planar configuration [57]. Such membrane structural rearrangement may enhance the binding reaction between the antigens and their respective antibodies. Studies have shown that pulse or DC electrical field exposure to various mammalian cells could alter cell membrane potentials and membrane structures [29-31], and could cause cell deformation [29-33]. Electrical fields could also possibly increase cell membrane permeability [32-34]. However, little has been done to study the effects of DEP on bacterial cells, and detailed mechanisms for the increase in immuno- reactivity of *Listeria *cells in DEP treatment still need further study.

None of the antibodies tested in this study displayed any significant decrease in the ELISA signals, suggesting that the use of DEP as a tool to manipulate bacterial cells in micro-devices or other biosensors would not affect the subsequent immuno-based detection of *Listeria*. On the contrary, DEP can sometimes enhance the signals of immuno-based detection using some antibodies, e.g. C11E9 and KPL antibodies to *Listeria *cells.

### DEP effects on cell growth profile in LCGM medium

A number of recently developed methods for rapid detection of bacteria are growth-based methods in which cell growth is a requirement of the detection procedure, such as impedance measurements [60,61], pH and conductivity dual measurements [42], and electrochemical measurements of oxygen consumption [62]. In these methods, regardless of the type of the signals, the detection or the quantification of bacterial concentration in a sample is based on the metabolic activity of the cells which produces a detectable signal. One of the most attractive advantages of these growth based methods is that they allow us to distinguish between viable and dead cells. Recent advances in microfabrication technologies have enabled scientists to fabricate micro-devices and have promoted these growth based methods to a more sensitive micro-chip-based stage. For example, a technique of "Impedance microbiology-on-a-chip" has been demonstrated by Gomez and coworkers [24]. The basic idea was to confine a few live bacterial cells into a small volume on the order of nano- to pico- liters, so that the metabolism of a few live cells in a low conductivity buffer could be rapidly detected by impedance measurement. DEP has been proven to be an effective approach to confine a few live cells into such micro-chips. However, this approach brought our attention to the possible effects of DEP treatment on cell growth profile in liquid medium.

**Figure 5:**
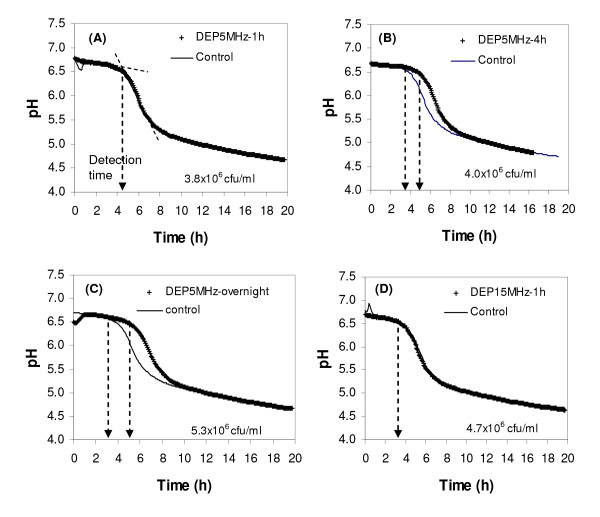
The growth profiles of DEP-treated *Listeria monocytogenes *in LCGM medium monitored by real time pH measurements, along with control samples. The DEP treatment conditions and initial cell numbers in the samples are shown in each plot. DEP voltages for all samples were at 20 Vpp. Arrows indicate the detection times on pH-growth curves.

Fig. 5 shows the pH-based growth curves of DEP-treated *Listeria *samples in LCGM medium, together with the growth curves of control samples. The BioV LCGM™ growth medium consisting of tryptone, yeast extract, glucose, and bovine serum albumin (BSA) was used in all the experiments for growth and recovery of stressed *Listeria *cells. This medium has its initial pH of 6.8 ± 0.2 and its initial conductivity of~1.2 ± 0.2 mS/cm. It has been proven to be a suitable medium for impedance or pH detection of bacterial growth in traditional settings and microdevices [24,42,43]. pH-based growth curves, pH as a function of growth time, have similar patterns to that of well studied impedance growth curves, and it is feasible to study the growth profiles of bacteria [42]. The pattern of the pH-based growth curve presents the typical sigmoid growth curve profiles for bacteria, including their lag phase, exponential growth phase and stationary phase. As shown in Fig. 5, for all pH-based growth curves, pH does not change in the first period which corresponds to the lag phase of the conventional enumeration based-bacterial growth phase. Significant pH drop (from~6.7 to~4.9) is observed when bacteria enter into the exponential growth phase. The pH of the medium becomes relatively stable again when bacterial growth reaches the stationary phase. The detection time (t_*D*_) (indicated by arrows in Fig. 5) at which the pH value starts to decrease significantly is correlated to the initial viable cell numbers in the medium. Therefore, for detection purposes, the cell number in the sample can be estimated by looking at the detection time which is inversely proportional to the initial cell number.

As shown in Fig. 5 (panel A), the *Listeria *cell sample treated by DEP at 5 MHz and 20 Vpp for 1 h has the same growth profile as the control sample, indicating that the 1h DEP treatment did not cause any significant change in cell growth profile in the LCGM medium. However, as seen in Fig. 5 (panel B and C), prolonged DEP treatments (4h and overnight) cause shifts in the pH-growth curves in comparison with those of control samples. The detection times of the samples treated with DEP for 4h or overnight were delayed by 1–2 h compared to the control samples without DEP treatments. The delays in the detection times implies that there were fewer viable cells or more damaged cells in the samples after prolonged (4h or longer) DEP treatment. The population of damaged cells may include the stressed cells, slow-growing cells, severely damaged and non-growing cells, and possibly dead cells. It is suspected that many membrane enzymes may absorb and transduce energy from the oscillating field during the exposure of cells to AC electrical fields [29]. This alteration may cause cell damage, cell stress, slow cell growth, and even cell death. Other possible alterations, such as cell membrane potential and structure changes, deformation, and increased membrane permeability, as mentioned above, may in combination affect cell growth. Studies have shown that exposure of 3T3 fibroblast cells to a high frequency field (1–40 MHz) extended cell cycle time from 18 h to 26 h [63,64]. We can also see in Fig. 5 (panel D) that the pH growth curve of the sample with DEP treatment at 15 MHz and 20 Vpp is the same as the control sample, indicating that the treatment of DEP at 15 MHz and 20 Vpp for 1 h did not cause any change in cell growth. These results suggest that the duration of DEP treatment, rather than the frequency of the DEP voltage, is a factor that induces the damages in cells. Therefore, DEP manipulation of bacterial cells for short durations (less than 1 h) is recommended when the cells will be analyzed by subsequent cell growth procedures.

### Cell viability on MOX plates

Table 1 shows the total viable cell counts on MOX plates for DEP-treated and -untreated *Listeria *cells. Four trials (all four samples were in the order of ~10^8 ^cfu/ml) were treated by DEP at 5 MHz and 20 Vpp for 4 h. The treated samples were diluted and appropriate dilutions were plated on MOX plates. The viable cell numbers of DEP-treated samples were compared with those of untreated samples. Decreases of ~56.8 – ~89.7% in viable cell number were observed for DEP treated samples, as shown in Table 1. MOX agar contains antibiotic agents which are introduced to suppress the growth of other competing microflora. However, injured or stressed *Listeria *cells are unable to grow in the presence of these selective agents to form characteristic colonies on MOX agar. Therefore, the discrepancy is due to the non-sustainability of some stressed/injured cells which could not grow on MOX agar. These results indicate that long duration of DEP treatment (5 MHz and 20 Vpp for 4 h) caused severe injury to cells which could not recover on MOX media. We did not observe significant changes in viable numbers on MOX plates for the samples with 1 h DEP treatments. The observations on MOX plating tests are consistent with the results we observed in the growth profile tests.

**Table 1:** Viable cell numbers of the tested samples before and after DEP treatment determined by plate counting on MOX plates, along with the calculated percentage differences.

**Sample**	**Cell number before DEP treatment (in 50 μl)**	**Cell number after DEP treatment (in 50 μl)**	**Percentage change**
1	(2.60 ± 0.99) × 10^8^	(6.30 ± 0.71) × 10^7^	-75.8%
2	(4.60 ± 0.61) × 10^8^	(4.70 ± 1.41) × 10^7^	-89.7%
3	(4.75 ± 1.27) × 10^8^	(2.05 ± 0.87) × 10^8^	-56.8%
4	(1.60 ± 0.41) × 10^8^	(3.30 ± 0.64) × 10^7^	-68.9 %

Cell numbers were averaged from colony counts on 3–5 plates.

DEP treatment: 5 MHz and 20 Vpp for 4 h.

It is believed that at such high frequency, the electrical field could penetrate to the interior of the cell as they experience positive DEP in DI water. It is reported that cells could be lysed by electroporation and/or electrofusion if the induced membrane potential exceeds ~1 V (Pethig, 1991). For cells with a radius of around 2.5–5 μm, an applied electrical field lower than about 1–3 × 10^5 ^V/m would not produce such effects [3]. In our experiments, the calculated maximum electrical field was about 8 × 10^5 ^V/m (20 V/25 μm), but we did not observe any cell lyses under the experimental condition, which may be due to the difference in cell types.

The results in growth profile tests and viability tests showed that long duration of cell exposure to DEP electrical fields could cause bacterial cell damage and affected cell growth and viability, which could induce undesirable effects on cell growth-based detection methods (delays in detection time, decreases in viable cell number). These findings suggested that experimental conditions of DEP should be taken into consideration to avoid or minimize negative effects on subsequent cell detection performance.

## Conclusion

In this study, we investigated the effects of DEP on the immuno-reactivity to different antibodies, growth profile, and cell viability. The immuno-reactivity tests using ELISA showed that the immuno-reactivity to KPL anti-*Listeria *antibodies and to C11E9 monoclonal antibodies was enhanced after DEP treatment, whereas the immuno-reactivity to other two antibodies (PAb Lm404 and PAb C639) showed no significant change. DEP treatment of 1 h (5 MHz or 15 MHz and 20 Vpp) caused no change in *Listeria *growth profiles in LCGM medium; however, longer treatment time (4 h or longer) did cause shifts in the pH-based growth curve, which was due to the reduced number of viable cells in the DEP-treated samples. It was found that 56.8–86.7% of cells in a population of 10^8 ^cfu/ml were injured by a 4 h DEP treatment at 5 MHz and 20 Vpp and were unable to form colonies on MOX selective agar plates. These results suggest that prolonged DEP treatment/operation for manipulating bacterial cells could produce negative effects on cell detection, particularly when it is used as a pre-concentration step prior to growth-based detection. For immuno-based detection, DEP manipulation may or may not affect the final detection signal depending on the type of antigen-antibody involved. It should be noted, however, that other alterations (from gene expression to cell morphology) may occur to DEP manipulated cells and may affect other biological functions of the cells; other factors such as medium conductivity and electrode materials may possibly affect the extent of DEP effects on cell functions; and these remained to be elucidated.

Biomedical and Biological Micro-Electro-Mechanical Systems (MEMS) technology has shown tremendous potentials in the development of new devices and sensors with scales and dimensions similar to biological species for a variety of applications in diagnostics, sensing and characterization of biological entities. DEP technique has been proven to be a powerful tool for manipulating biological entities in such engineering microfabricated microdevices. The integration of DEP will advance the performance of various biosensors, microfluidic chips, and lab-on-a-chip devices by providing multi-functions such as cell concentration, separation or sorting, and enhancement of immunoreaction in these microdevices. This study brings attention to the need for careful selection of DEP conditions for manipulation of biological entities to avoid or minimize possible negative effects on biological detection in DEP integrated microdevices.

## Competing interests

The authors declare that they have no competing interests.

## Authors' contributions

LY and PPB contributed to the design of the study, the acquisition and analysis of data, and the preparation of the manuscript. AKB and RB contributed to the design and coordination of the study and preparation of the manuscript. All the authors read and approved the final manuscript.
